# Partial Loss of NEMO Function in a Female Carrier with No Incontinentia Pigmenti

**DOI:** 10.3390/jcm14020363

**Published:** 2025-01-09

**Authors:** Cristina Cifaldi, Mayla Sgrulletti, Silvia Di Cesare, Beatrice Rivalta, Agolini Emanuele, Lucia Colucci, Giusella Maria Francesca Moscato, Marta Matraxia, Chiara Perrone, Gigliola Di Matteo, Caterina Cancrini, Viviana Moschese

**Affiliations:** 1 Department of Systems Medicine, University of Rome Tor Vergata, 00133 Rome, Italy; cristina.cifaldi@uniroma2.it (C.C.); gius_va@virgilio.it (G.M.F.M.); di.matteo@med.uniroma2.it (G.D.M.); cancrini@med.uniroma2.it (C.C.); 2 Pediatric Immunopathology and Allergology Unit, Policlinico Tor Vergata, University of Rome Tor Vergata, 00133 Rome, Italy; maylasg@gmail.com; 3 PhD Program in Immunology, Molecular Medicine and Applied Biotechnology, University of Rome Tor Vergata, 00133 Rome, Italy; 4 Unit of Clinical Immunology and Vaccinology, IRCCS Bambino Gesù Children Hospital, 00165 Rome, Italy; di.cesare@med.uniroma2.it; 5 Research Unit of Primary Immunodeficiency, IRCCS Bambino Gesù Children Hospital, 00165 Rome, Italy; beatrice.rivalta@opbg.net (B.R.); lucia.colucci@live.it (L.C.); 6 Laboratory of Medical Genetics, Translational Cytogenomics Research Unit, Bambino Gesù Children Hospital IRCCS, 00165 Rome, Italy; emanuele.agolini@opbg.net (A.E.); marta.matraxia@opbg.net (M.M.); chiara.perrone@opbg.net (C.P.)

**Keywords:** NEMO deficiency, incontinentia pigmenti, female carrier, immune dysregulation

## Abstract

**Background/Objectives**: The nuclear factor (NF)-kB essential modulator (NEMO) has a crucial role in the NFκB pathway. Hypomorphic *IKBKG* pathogenic variants cause ectodermal dysplasia with immunodeficiency (EDA-ID) in affected males. However, heterozygous amorphic *IKBKG* variants could be responsible for Incontinentia Pigmenti (IP) in female carriers. Typically, IP patients do not exhibit immunodeficiency, although hypomorphic variants might lead to immunodeficiency in female IP patients. Here, we report the case of an *IKBKG* female carrier, with no IP but an unexpected picture of immunodeficiency. She had a positive family history for the same genetic condition. **Methods**: We performed immunological, molecular, and functional analysis to evaluate NEMO contribution. **Results**: The patient was healthy until the age of 25 when severe asthma and Hashimoto thyroiditis occurred. She had HLAB27-positive ankylosing spondylitis, non-tubercular mycobacteriosis, and pulmonary aspergillosis infections. We found CD19+ B cell lymphopenia and T cell subset alterations. Sanger sequencing revealed a heterozygous *IKBKG* variant at position +1 of the 5′ UTR of the gene which disrupted the normal pre-mRNA splicing. We observed a decreased NEMO protein expression, a reduced level of mRNA, and a defective NF-κB pathway. **Conclusions**: These findings suggest a possible correlation between the partial loss of NEMO function and the immunodeficiency observed in this patient. This case could expand our understanding of NEMO deficiency in female carriers.

## 1. Introduction

The nuclear factor (NF)-kB essential modulator (NEMO), also known as IKK-g, is the regulatory subunit of the IKK (Inhibitor of kB Kinase) complex that also comprises IKK-α and -β. Indeed, it plays a pivotal role in tissue homeostasis by transmitting extracellular or intracellular signals and controlling NF-kB-regulated genes [1]. Upon stimulation, the IKK complex phosphorylates the inhibitor of kappa B (IkB), leading to its degradation and facilitating the nuclear translocation of NF-κB and the transcription of genes involved in inflammation, immunity, and cell survival [2].

The complete loss of function (LoF) of NEMO is lethal at the embryonic stage in males, possibly through enhanced sensitivity to apoptosis, especially in the liver, which might be partly due to the missing survival function of NF-kB and partly due to an NF-kB-independent function, preventing uncontrolled RIPK1-dependent apoptosis [3].

Hypomorphic hemizygous missense variants or short truncations of *IKBKG* in males that impair but do not abolish NF-kB signaling lead to hypohidrotic ectodermal dysplasia with immunodeficiency (EDA-ID, OMIM#300291) as well as immunodeficiency without EDA [4,5,6,7,8]. Patients with EDA-ID present impaired development of skin appendages characterized by poor scalp hair, rare conical teeth, a lack of sweat glands (hypohidrosis), and frontal bossing [9].

The immunodeficiency derives from impaired T and B cell development and function with dysregulated immunoglobulin synthesis or hyper–immunoglobulin M (hyper-IgM) syndrome, defective anti polysaccharide antibody synthesis (anti-pneumococcal antibody and isohemagglutinin), and reduced lipopolysaccharide (LPS) and IL-1 family protein responses. Also, susceptibility to bacterial (especially pyogenic and mycobacteria), viral, and fungal infection due to impaired Toll-like receptor function and defective natural killer (NK) cell activity can be observed [6,7,10,11,12,13,14,15,16].

Females carrying an LOF variant of *NEMO* survive because of skewed X-chromosome inactivation (XCI) [17] favoring the WT *NEMO* allele and present Incontinentia Pigmenti (IP, OMIM#308300). IP females show an unbalanced X-chromosome inactivation mosaicism as a consequence of the survival of cells expressing the WT *NEMO* allele. Conversely, the mutant allele *NEMO*-expressing cells are prone to death, at least in peripheral blood and skin tissues, as commonly tested. Most IP female patients have shown a recurrent deletion of the *IKBKG* gene that encompasses exon 4-10, leading to a truncated protein and despite this rearrangement, clinical symptoms might be absent. This type of variant is generally lethal in males, apart from cases with Klineferter syndrome (47, XXY karyotype) [18,19,20,21,22,23,24] or mosaicism [25,26].

Generally, IP disease in females presents with a broad heterogeneous phenotype affecting the skin, teeth, nails, hair, and eyes. In addition, IP is always associated with persistent inflammatory response that occurs in the neuroectodermal tissues during development or very soon after birth (2 weeks after birth). Also, IP is associated with autoimmunity [27,28,29,30,31,32].

Because of the selective death of NEMO-deficient cells in female carriers, the mutated *NEMO* or the absence of protein is undetectable in the tissues cited above. Accordingly, the identification of the disease-causing variants (missense or splice) on the NEMO mRNA is unlikely by cDNA sequencing methods [33].

Here, we report the case of a female carrier of a splice *IKBKG* variant, with no IP but an unexpected infectious and immunodysregulatory phenotype. She had a positive family history for the same genetic condition. These findings suggest a possible correlation between the partial loss of NEMO function and the inflammatory trait observed in this patient. This unique case could expand our understanding of NEMO deficiency in female carriers.

## 2. Materials and Methods

### 2.1. Ethics and Informed Consent

All procedures performed in the study were in accordance with the ethical standards of the institutional research committee and with the 1964 Declaration of Helsinki and its later amendments. Informed consent, following standard ethical procedures, was obtained from the case-index patient and her parents. This study was approved by COMITATO ETICO INDIPENDENTE AZIENDA OSPEDALIERO UNIVERSITARIA POLICLINICO TOR VERGATA, Viale Oxford 81, 00133 Rome, Italy (protocol code 59/16; date of approval: 18 March 2016).

### 2.2. Molecular Studies

#### 2.2.1. Trio-Based WES Analysis

DNA was extracted from peripheral blood (PB) with QIAgen columns (QIAsymphony DNA minikit, Qiagen, Hilden, Germany) according to the manufacturer’s instructions and quantified using an ND-1000 spectrophotometer (NanoDrop; Thermo Scientific, Waltham, MA, USA) and an FLx800 Fluorescence Reader (BioTek, Winooski, VT, USA). According to the manufacturer’s protocol, we performed WES using the Twist Human Core Exome Kit (Twist Bioscience, 681 Gateway Blvd, South San Francisco, CA, USA). Then, we sequenced on the Illumina NovaSeq6000 platform (Illumina, Inc., San Diego, CA, USA). We aligned reads to human GRCh37/UCSC hg19 and used the BaseSpace pipeline and the Geneyx software (version 6.0) LifeMap Sciences, respectively, for the variant calling and annotating variants. The variants were filtered by in silico analysis using public databases (dbSNP, Combined Annotation Dependent Depletion (CADD) V.1.3, Polymorphism Phenotyping v2 (PolyPhen-2), Sorting Intolerant from Tolerant (SIFT), and Mutation Taster). Global minor allele frequency (MAF) for analyzed variants was calculated according to the Genome Aggregation Database (gnomAD). The variants were evaluated by VarSome and categorized in accordance with the ACMG recommendations. Variants were also examined for coverage and Qscore (minimum threshold of 30) and analyzed by the Integrative Genome Viewer (IGV) version 2.16.1.

#### 2.2.2. Sanger Sequencing

The exons of the *IKBKG* (NCBI NM_001099857.5) gene were amplified by PCR (GoTaq Polimerase-Promega) and sequenced using the BigDye Terminator version 3.1 Cycle Sequencing Kit (Life Technologies, Milan, Italy) on an ABI PRISM 3130 (Life Technologies, Milan, Italy).

#### 2.2.3. cDNA

Total RNA was isolated from 3 × 10^6^ lymphoblastoid cell lines (LCLs) of the patient, her mother, and HD by the Trizol procedure (T9424, Sigma-Aldrich, Saint Louis, MO, USA). Reverse transcription was performed using SuperScript™ III First-Strand Synthesis SuperMix reaction kit (Life Technologies, Milan, Italy) in conformity with the manufacturer’s instructions, with distinct overlapping primer pairs (available on request) to amplify the cDNA.

#### 2.2.4. Real Time-PCR

The Sybr Green expression assay was performed using a LightCycler^®^ 96 (Roche Diagnostics, Mannheim, German). The manufacturer’s instructions were followed for PCR conditions. We set up PCR with ATGAATAGGCACCTCTG Sense and AGAATCTGGTTGCTCTG AntiSense primers (melting temperature 56 °C) and 2.5 × Real Master Mix = 20 × SYBR solution in a total volume of 25 mL. The standard curves were derived from the 8, 16, and 32 ng amplification of a control cDNA. The target amplicons absolute quantification (AbsQ) was performed by interpolating the threshold cycle number (Ct) against the corresponding standard curve. We assumed that the rate changes in Ct are identical for β-Actin and NEMO genes. The control was normalized to value 1.2.2.5. X chromosome inactivation: DNA was pre-digested with a methylation-sensitive restriction endonuclease (HpaII, Life Technologies, Milan, Italy), whose binding site is located in proximity of short polymorphic tandem repeats on the androgen receptor (AR) gene. Digested DNA samples were then amplified by PCR (AmpliTaq Gold DNA Polymerase, Life Technologies, Milan, Italy), and the products were analyzed using an electrophoretic run on automatic sequencer ABI 3500xL (Applied Biosystem, Milan, Italy) and LightCycler® 96 software (version 1.1.0.1320, Roche Diagnostics, Mannheim, Germany).

### 2.3. Western Blot

Peripheral Blood Mononuclear Cells (PBMCs) and LCLs from the patient, her mother, and HD were incubated with complete JS 1× lysis buffer (50 mM Tris/HCl ph 8, 1.5 mM MgCl_2_, 150 mM NaCl, 5 mM EDTA, 1% Triton-X, 10% glycerol, 1 mM PMSF, aprotinin 1 mg/mL, pepstatin 1 mg/mL, leupeptin 1 mg/mL, and 1 mM DTT) for 20 min in ice and centrifugated at 1600 rpm for 10 min at 4 °C to obtain total protein lysates. The lysates were size-fractionated by SDS-PAGE 10% gel and then transferred to nitrocellulose membrane (Protran by Schleicher & Schuell-Bioscience). The membrane was blocked in 5% BSA at room temperature and then incubated with the NEMO/IKKγ (FL-419) (sc-8330 Santa Cruz Biotechnology, Inc., Heidelberg, Germany) at 4 °C o.n, washed, and incubated with goat anti-rabbit IgG (1 h, 1:5000). β-actin (1 h, 1:5000, T9424, Sigma-Aldrich, Saint Louis, MO, USA) was used as housekeeping.

### 2.4. Peripheral Blood Immunophenotype

Flow cytometric analyses were performed on EDTA blood samples within 24 h of venipuncture. After the lysis of red blood cells with ammonium chloride (NH_4_Cl), lymphocytes were washed, resuspended in PBS, and stained to identify T and B cell subsets with the following mouse anti-human antibodies: CD45RA (clone T6D11; Miltenyi Biotec, Bergisch Gladbach, Germany), CD3 (clone BW264/56; Miltenyi Biotec, Bergisch Gladbach, Germany), CD4 (clone OKT4; Becton Dickinson, Milan, Italy), CD27 (clone M-T271; Becton Dickinson), TCR α-beta (clone T10B9; Becton Dickinson, Milan, Italy), TCR gamma-delta (11F3; Miltenyi Biotec, Bergisch Gladbach, Germany), CD8 PE- (clone RPA-T8; Becton Dickinson, Milan, Italy), CCR7 (clone 3D12; Life Technologies, Milan, Italy), CD19 (clone SJ25C1; Becton Dickinson, Milan, Italy), CD16 (clone 3G8, Life Technologies, Milan, Italy), CD56 (clone MY31; Becton Dickinson, Milan, Italy), CD21 (clone B-ly4; Becton Dickinson, Milan, Italy), IgD (clone IA6-2; Becton Dickinson, Milan, Italy), CD24 (clone ML5; Becton Dickinson, Milan, Italy), Goat F(ab)2 anti-Human IgM(μ) (Jackson ImmunoResearch. Cambridge House, UK), and CD38 (clone HIT2; Becton Dickinson, Milan, Italy). Cells were incubated with the specific antibody cocktail for 30 min at 4 °C, washed, and resuspended in PBS for acquisition. At least 50,000 events were acquired within the lymphocyte gate. Data were acquired on a FACSCanto II (Becton Dickinson, Milan, Italy) and analyzed with FlowJo software (Tree Star Inc., version 9.3.2, Ashland, OR, USA).

### 2.5. Generation of LCLs

PBMCs were isolated from patients III.2, her mother II.2, her cousin III.3, and HD by density gradient centrifugation (Ficoll-Paque PLUS, GE Healthcare, Milan, Italy) and washed in PBS. After isolation, PBMCs were resuspended in complete RPMI in 20% of FBS (plus 100 U/mL penicillin and streptomycin and 2 mM l-glutamine) with B95-8 EBV supernatant (0.22 μm filtered) for 2 h (37°C/5% CO_2_) and then plated. The day after, 5 mg/mL cyclosporine in 1 mL of 20% FBS was added. Two weeks were required to generate LCL clumps.

#### 2.5.1. NEMO Expression

LCLs (250.000 per tube) were washed with FACS buffer and incubated with Fc-block (553141, BD Biosciences, Milan, Italy) for 5 min at RT to block the receptors. Then, cells were washed twice, permeabilized for 30 min at RT, following the manufacturer’s protocol, and centrifuged 1600 rpm for 5 min. After two washes with FACS buffer, the cells were intracellularly stained with anti-monoclonal NEMO/IKKγ (FL-419) (sc-8330, Santa Cruz Biotechnology, Inc., Heidelberg Germany) for 30 min at RT, washed, and stained with Goat-anti-Rabbit IgG (ab6717, Abcam, Prodotti Gianni s.r.l, Milan, Italy) for 30 min at RT. Data were collected with FACS Canto II (Becton Dickinson, Milan, Italy). Data were analyzed with FlowJo software (Tree Star Inc., version 9.3.2, Ashland, OR, USA) and with Graph-Pad Prism, version 6.2 (Graph Pad Software, La Jolla, CA, USA).

#### 2.5.2. IκBα Degradation

PBMCs were isolated by density gradient centrifugation using Ficoll-Paque PLUS (GE Healthcare, Milan, Italy), washed in PBS, and preserved in complete RPMI (Sigma-Aldrich, Saint Louis, MO, USA) containing 10% FBS, plus 100 U/mL penicillin and streptomycin (Sigma-Aldrich) and 2 mM l-glutamine. In total, 3 × 10^5^ PBMCs per tube were stimulated with phorbol 12-myristate 13-acetate (PMA 100 ng/mL) and Ionomycin (1 μg/mL) at 37 °C for 0, 15, 30, and 60 min. The cells were washed with FACS buffer, then incubated with Fc-block (553141, BD Biosciences, Milan, Italy) for 5 min at RT, and stained with anti-CD4+ and CD19+ antibodies on ice for 30 min at 4 °C. Then, the cells were washed twice, permeabilized for 30 min at RT, and washed twice with FACS buffer. For intracellular staining, the cells were incubated with anti-monoclonal IкBα (H-4) (sc-1643, Santa Cruz Biotechnology, Inc., Heidelberg Germany) for 30 min at RT, washed, and stained with anti-Mouse IgG (11-4011-85, Life Technologies, Milan, Italy). After two washes, data were collected with FACS Canto II (Becton Dickinson, Milan, Italy). All data were analyzed with FlowJo software (Tree Star Inc., version 9.3.2, Ashland, OR, USA) and with Graph-Pad Prism, version 6.2 (Graph Pad Software, La Jolla, CA, USA)

#### 2.5.3. Dihydrorhodamine (DHR)

Heparinized blood samples were processed within 2 h after venipuncture. In total, 10 μL of opsonized *E. Coli* or PMA was added to 50 μL of the blood samples and gently vortexed; after 10 μL of DHR123 was added, samples were gently vortexed and incubated for 20 min at 37 °C. Then, the blood samples were treated with lysis solution, vortexed, and incubated for 5 min at room temperature. After incubation, 1 mL of deionized water was added and after 10 min, samples were collected within 2 h with FACS Canto II (Becton Dickinson) and analyzed with FlowJo software (Tree Star Inc, version 9.3.2) and Graph-Pad Prism, version 6.2 (Graph Pad Software, La Jolla, CA, USA). All procedures were conducted using FagoFlowEx kit (cat ED7042, Exbio, Vestec, Czech Republic).

#### 2.5.4. Cytokine Assays

PBMCs were isolated by density gradient centrifugation using Ficoll-Paque PLUS (GE Healthcare, Milan, Italy), washed twice in PBS, and resuspended in complete RPMI containing 10% FBS, plus 100 U/mL penicillin and streptomycin and 2 mM l-glutamine (Sigma-Aldrich, Saint Louis, MO, USA). PBMCs per tube (2.5 × 10^5^) were stimulated with phorbol 12-myristate 13-acetate (PMA 25 ng/mL) and Ionomycin (1 μM/mL) at 37 °C o.n for IFNγ production, with lipopolysaccharide (LPS) (200 ng/mL) or PMA (200 ng/mL) at 37 °C for 12 h, for TNFα and IL-8 production, in the presence of the secretion inhibitor Brefeldin A (BFA: 10 ngr/mL) (Sigma-Aldrich, Saint Louis, MO, USA). After stimulation, the cells were washed with FACS buffer and incubated for surface staining with anti-CD3 (clone BW264/56; Miltenyi Biotec, Bergisch Gladbach, Germany) and anti-CD45RA (clone T6D11; Miltenyi Biotec, Bergisch Gladbach, Germany) for 15 min at 4 °C. After two washes, the cells were fixed for 20 min at RT and then permeabilized, according to the manufacturer’s protocol, and washed twice with FACS buffer. The cells were then stained with anti-IFNγ (Clone ReaA600; Miltenyi Biotec, Bergisch Gladbach, Germany), TNFα (Clone MAb11; Biolegend, Milan, Italy), or IL-8 (BD). IFNγ expression was detected gating on CD3+ CD45RA- T cell, whereas TNFα and IL-8 production was measured after gating on monocytes.

## 3. Results

### 3.1. Case Description and Family History

The patient is a Caucasian 37-year-old female, born to non-consanguineous Italian parents, with positive family history for immunodeficiency. She was identified as a female carrier of a splice *IKBKG* variant at the age of 23, following the identification of the *IKBKG* hypomorphic variant in her two maternal cousins who presented with a severe infectious phenotype and no ectodermal dysplasia [34]. Her first maternal cousin, a 26-year-old male, had experienced severe, recurrent upper and lower respiratory tract infections, recurrent diarrhea, and isolated hematuria since the age of 3. Immunological evaluation at age 4 revealed humoral and cellular immune deficiencies, characterized by elevated IgA levels with normal IgG and low IgM, normal antibody responses to rubella, waning specific antibody responses to tetanus and pneumococcus, and no specific antibody response following hepatitis B, measles, and mumps immunization. Although standard immunophenotype was normal, extensive analysis found low switched memory B cells and decreased in vitro T-cell proliferation to tetanus toxoid antigen.

Since age 10, he has been on immunoglobulin replacement therapy, with good infectious control. However, over a ten-year period, he developed chronic intestinal and liver inflammation and severe bronchiectasis. The second maternal cousin, a 20-year-old male, has suffered from recurrent upper and lower respiratory tract infections since childhood. His first immunological work-up identified borderline IgM and normal IgA levels that both declined over time, with poor specific antibody response to pneumococcus. He also showed low switched memory B cells. Since the age of 8, he has been on immunoglobulin replacement therapy with partial control of infections. Chronic nephropathy and cholestatic liver disease both with inflammatory and granulomatous features, idiopathic hypereosinophilic syndrome, and bronchiectasis complicated his outcome. The patient’s brother, who had no features of ectodermal dysplasia, presented with recurrent and severe respiratory infections since early childhood and was later diagnosed with CVID. He then developed disseminated Mycobacterium avium intracellular infection with worsening and recurrent bouts of pneumonia, chronic diarrhea, and malnutrition. He died at the age of 19 years due to widespread Mycobacterium avium infection. Post-mortem genetic testing, performed after the diagnosis of his maternal cousins, identified the same *IKBKG* mutation. Furthermore, the patient’s mother and maternal aunt are asymptomatic carriers of this condition. 

**Figure 1:**
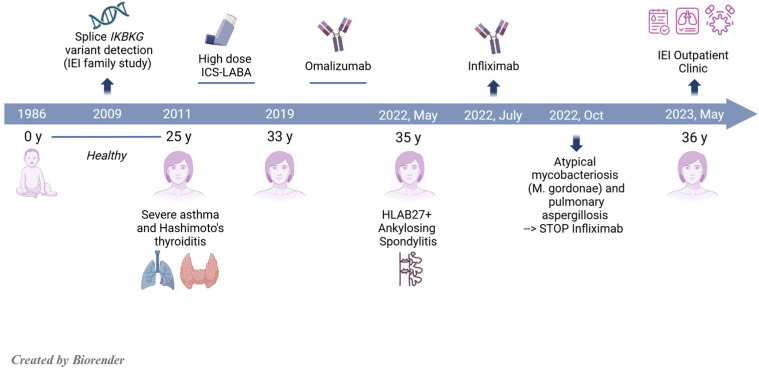
Timeline showing patient’s clinical and treatment history.

As detailed in Figure 1, the patient was healthy until the age of 25 when severe asthma and Hashimoto thyroiditis occurred. At the age of 33, omalizumab was initiated after the patient failed to respond to high-dose inhaled corticosteroid-long-acting beta-agonist (ICS-LABA) therapy for asthma control. At the age of 35, HLAB27-positive ankylosing spondylitis was diagnosed, and the patient was promptly treated with infliximab, due to the lack of response and poor tolerance to nonsteroidal anti-inflammatory drugs (NSAIDs) and cyclooxygenase-2 inhibitors (Coxibs). In the past, the patient received neither systemic corticosteroids nor standard disease-modifying anti-rheumatic drugs (DMARDs). Infliximab was interrupted after three months due to non-tubercular mycobacteriosis (*M. gordonae*) and pulmonary aspergillosis, requiring 1-year-long specific antimicrobial therapy. Seven months after infliximab interruption, she was referred to our Immunology Clinic for consultation. Immunological assessment revealed findings partially overlapping with those observed in the other family members. In detail, high serum IgA (392 mg/dL) and a mild IgG2 subclass deficiency (25 mg/dL) were detected, with increased IgE (208 UI/mL) and adequate specific antibody response to pneumococcus (pneumococcal antibody 39.55 mg/dL, protective from 7 mg/dL). The patient is currently on omalizumab, with good asthma control. Physical rehabilitation and analgesic therapy are used to control symptoms related to ankylosing spondylitis. JAK inhibitor therapy is currently postponed due to the risk of mycobacterial reactivation.

### 3.2. Immunological Assessment

Lymphoid cell distribution revealed a CD19+ B cell lymphopenia (5%) with a reduction in CD24^++^CD38^++^ transitional B cell percentage (0.7%). A significantly increased proportion of CD4^+^CD27^+^CD45RA^−^ central memory T cells (43%), combined with slightly decreased effector memory CD4+CD27^−^CD45RA^−^ (10%) and CD8^+^CCR7^+^CD45RA^−^ central memory T cells (2.2.%), was found. The frequencies of regulatory T cells (CD4^+^ CD25^+^CD127^low^FOXP3^+^), Th17 cells (CD4^+^CD45RA^−^CXCR5^−^CCR6^+^), and circulating CD4^+^CD45RO^+^CXCR5^+^ follicular helper T-cells were in the normal ranges. Immunological data are reported in Table 1.

### 3.3. Molecular Analysis

**Figure 2:**
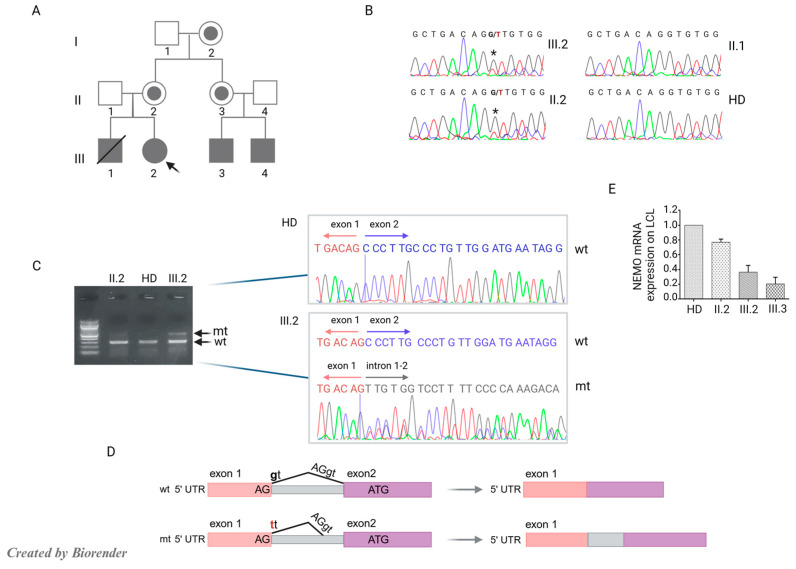
Characterization of NEMO-III.2. (**A**) Family pedigree showing proband III.2 (black arrow), her carrier mother II.2, her brother III.1, and cousins III.3 and III.4 affected by NEMO deficiency. (**B**) Sanger sequencing performed on III.2, II.1, II.2, and HD genomic DNA showing the NG_009896:g.10503G>T variant indicated by “*”. (**C**) cDNA analysis of *IKBKG* fragments obtained from III.2 and II.2. Black arrows indicate the wt and wt + mt sequences of II.2 and III.2, respectively, on the agarose gel. Chromatograms showing the NG_009896:g.10503G>T variant causing the retention of the intron between exon 1 and 2 in III.2 compared to HD. (**D**) Schematic of the normal (upper panel) and alternative splicing of *IKBKG* exon 1 and 2 detected in III.2. (**E**) Real-time PCR analysis showing reduced NEMO mRNA levels, normalized to β-Actin, in LCLs from II.2, III.2, III.3, and HD. The graph represents the mean of two experiments.

Sanger sequencing, performed in the patient and her mother, confirmed the heterozygous *IKBKG* variant (NG_009896: g.10503G>T; rs2148357351) (Figure 2A,B) already reported in two maternal cousins [34]. This mutation led to a G to T substitution at position +1 of the donor splice site in the 5′ untranslated region (UTR) of the *IKBKG* gene. Whole-exome sequencing (WES) was performed in the index patient (III.2) to identify potential additional genetic aberrations. We did not detect any suggestive variant in other genes including those related to Mendelian susceptibility to mycobacterial diseases (MSMD). Among the genetic findings, WES identified two heterozygous missense variants in genes related to autosomal recessive diseases. The first one of uncertain significance (VUS) in the *POLE* gene (c.2453A>G p.Tyr818Cys) and the second one likely pathogenic in the *MASP2* gene (c.1187G>A p.Cys396Tyr), both segregated from the healthy father (Table S1 Supplementary).

X-chromosome inactivation performed on the patient’s DNA showed a random inactivation. Further analysis conducted on cDNA derived from the patient’s and her mother’s (II.2) PBMCs revealed the presence of one wild-type (wt) product and a second alternative product in which the G>T substitution has disrupted the normal exon 1 to exon 2 splice junction generating a cryptic splice site in the intron between exon 1 and 2. This modification caused the retention of part of the intron, resulting in at least one alternatively spliced mRNA product in the patient, as illustrated in Figure 2C,D. Based on cDNA findings, we measured the wt NEMO mRNA levels using real-time PCR in LCLs derived from the patient, her mother (II.2), her cousin (III.3), and HD. We used β-Actin mRNA levels as an internal control. The patient’s and III.3’s LCLs exhibited 2.3- and 7.1-fold lower NEMO mRNA levels, respectively, while II.2 had 1.25-fold lower levels than HD (Figure 2D).

### 3.4. Functional Investigations

**Figure 3:**
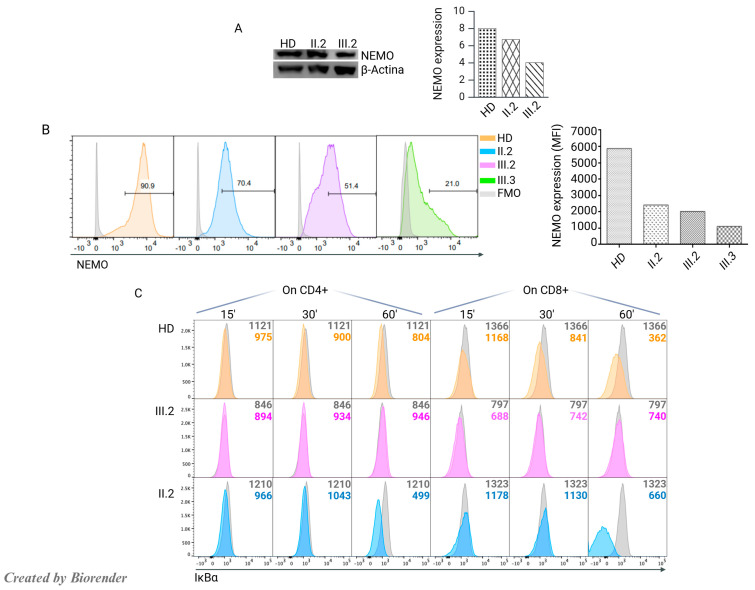
Functional analysis of NEMO. (**A**) NEMO protein expression by WB performed on PBMCs derived from III.2, II.2, and one HD showing reduced protein expression in III.2 and a slight reduction in II.2. (**B**) NEMO protein expression by FACS on LCLs from one HD, II.2, III.2, and the NEMO-pt III.3. The gray histograms represent FMO, the orange histograms represent HD, the blue histogram represents the mother’s cells, the pink histogram shows reduced expression in III.2, and the green histogram represents nearly absent NEMO expression in III.3; the right graph shows the MFI. (**C**) Representative plots of IkBα degradation in CD4^+^ and CD8^+^ T cells from III.2, II.2, and HD. The numbers inside the plots represent the MFI. The gray histograms show IkBα expression at time 0 without stimulation. The colored histograms show IkBα degradation after stimulation with PMA/ionomycin.

We investigated the effect of the g.10503G>T variant on NEMO expression. Protein analysis, conducted on the PBMCs from the patient, II.2, and HD as depicted in Figure 3A, revealed a decrease in NEMO protein expression in the patient III.2 and, a slight reduction in her mother II.2. These findings were further confirmed by flow-cytometry on LCLs derived from II.2, III.2, her cousin III.3 affected by NEMO deficiency, and HD, as shown in Figure 3B.

To determine the impact of the *IKBKG* variant on the signaling pathway, we evaluated IκBα degradation at different times after PMA/Ionomycin stimulation. An impairment of IκBα degradation was confirmed in patient CD4^+^ and CD8^+^ T cells in comparison to HD and II.2 cells (Figure 3C). This result clearly demonstrated a defective NF-κB pathway.

**Figure 4:**
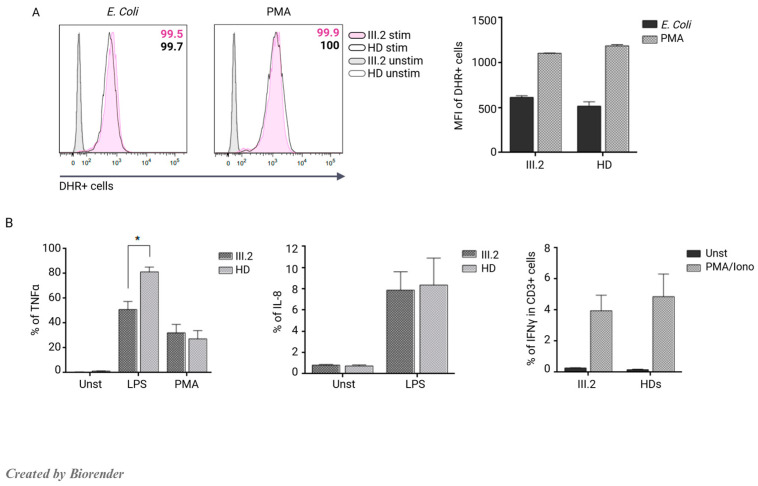
Functional characterization of III.2. (**A**) Evaluation of NADPH oxidase activity on neutrophils showing normal respiratory burst. Filled gray histograms represent unstimulated III:2 neutrophils, empty gray histograms represent unstimulated HD neutrophils, filled pink histograms represent stimulated III.2 neutrophils, and empty black histograms represent stimulated HD neutrophils. The right graph shows the MFI (mean ± SEM of n = 2 experiments). (**B**) Monocytes’ cytokines. The left panel shows decreased monocytes’ TNFα production *p* value (*) 0.0244 (mean ± SEM of n = 2 experiments), the middle panel shows normal monocytes’ IL-8 production (mean ± SEM of n = 2 experiments), and the right panel displays decreased IFNγ production from CD3^+^CD45RA^−^ memory T cells (mean ± SEM of n = 2 experiments) after indicated stimulations.

We also assessed the activity of nicotinamide adenine dinucleotide phosphate (NADPH) oxidase using a DHR assay to better address BCG infection. We observed normal respiratory burst by neutrophils of III.2 and levels of reactive oxygen species (ROS) production comparable to those of HD after stimulation with either opsonized *E.Coli* or PMA (Figure 4A). In addition, we tested monocytes’ capacity to produce proinflammatory cytokines such as Tumor Necrosis Factor alpha (TNFα) or interleukin 8 (IL-8).

Compared to HD, the patient’s monocytes exhibited a significant 1.59-fold lower TNFα production after LPS stimulation and normal IL-8 levels. Finally, we observed a 1.2-fold lower IFNγ production by CD3^+^CD45RA^−^ T cells after PMA/ionomycin stimulation (Figure 4B) than HD.

## 4. Discussion

Incontinentia Pigmenti is a rare neuroectodermal disorder that is observed in female carriers with heterozygous pathogenic variants in the *IKBKG* gene. It is an X-linked dominant disorder and has a multisystemic and developmental phenotype. In the male fetus, it is usually lethal due to the tumor necrosis factor α (TNFα)-induced cell death of keratinocytes [35,36]. However, random or skewed X-chromosome inactivation allows for female survival with various disease severity. While immune function is typically normal in patients with IP, hypomorphic *IKBKG* variants can lead to immunodeficiency similar to that which occurs in males [37].

We report a unique case of a female patient with NEMO deficiency without IP that expands our understanding of this disorder.

Our patient was healthy until the age of 25 when she suffered from severe asthma and Hashimoto thyroiditis; then, she developed HLAB27-positive ankylosing spondylitis, atypical mycobacteriosis, and pulmonary aspergillosis over time.

Interestingly, some male patients harboring *IKBKG* variants have been reported to exhibit immunodeficiency characterized by diminished IFN-γ and IL-12 secretion by PBMCs in response to PHA- or CD3-specific antibodies, with no ectodermal disorders as in EDA [6,38,39,40,41]. Moreover, specific missense *IKBKG* variants can lead to susceptibility to mycobacterial infections with no developmental defects, in the presence of a normal amount of NEMO mutant protein [38]. This susceptibility arises from *NEMO* hypomorphic variants which compromise T cell-dependent, CD40-dependent IL-12 production in the myeloid cells of affected individuals. Similarly, IP females with abnormalities of the immune system due to random X inactivation can also present mycobacterium infection [42,43]. Other pathways required to control mycobacterial infections involve tumor necrosis factor alpha (TNF-α), which plays a key role in granuloma formation. This process is critical to control bacterial spreading throughout the body [44,45,46]. Of note, the patient developed a mycobacterial infection after receiving infliximab therapy, which significantly suppresses cellular immune responses to Mycobacterium tuberculosis [47,48,49]. Therefore, we cannot establish whether mycobacteriosis and pulmonary aspergillosis were secondary to the immunosuppression following infliximab treatment rather than resulting from the primary impairment of NF-κB signaling. Therefore, clinical and molecular screening and close microbiological monitoring should be considered by a rheumatologist in patients with a family history of inborn errors of immunity (IEIs).

As NF-κB is involved in the immune and inflammatory responses and protection against apoptosis, a loss of function or absence of the *IKBKG* gene function contributes to impaired or null NF-κB activity, leading to IP due to increased susceptibility to apoptosis of *IKBKG*-deficient keratinocytes [50]. Indeed, the apoptotic *IKBKG*-deficient keratinocytes in female carriers release danger-associated molecular patterns (DAMPs), lysophoshatidylcholine (LCP), nucleotide ATP/AU, and Tumor Growth Factor (TGFβ) to adjacent keratinocytes expressing the wt form of *IKBKG* and evoke signals of NF-κB activation and immune-inflammatory responses such as chemokine and cytokine production released by T cells, NK, monocytes, and macrophages [51]. The recruitment of eosinophils, which undergo degranulation and the release of proteases that degrade adhesion molecules between keratinocytes, leads to the first stage of clinical manifestation in IP patients [52,53].

It is plausible that the hypomorphic variant of our patient allows residual protein expression and adequate NEMO signaling, which in turn prevents the initiation of keratinocyte-derived inflammatory processes and the development of IP. On the other hand, the clinical phenotype is widely variable in IP patients and the genotype–phenotype correlation is not clearly straightforward.

In the described patient, the G to T substitution at position +1 of the donor splice site in the 5′ UTR of the *IKBKG* gene led to abnormally spliced NEMO mRNA species with 2.3-fold decreased levels of wt NEMO mRNA and a 2.1-fold lower expression level of the NEMO protein compared to normal control. Moreover, our index patient had reduced, albeit present, protein expression that we suggest correlates to an altered IκBα degradation and a mild, albeit significant, impairment of TNFα along with a moderate reduction in IFNγ production.

Her healthy mother (II.2), while presenting a similar, although less evident, reduced NEMO expression, showed an IκBα degradation comparable to the healthy control. We can hypothesize that in our patient, the residual signaling from the NEMO protein is inadequate for normal immune function but is sufficient to prevent IP [36]. On the other hand, the residual NEMO activity could be able to prevent apoptosis and the release of proinflammatory signals, opening a novel perspective on NEMO function. We cannot exclude that the ankylosing spondylitis observed in our patient could also be a consequence of the altered NF-κB signaling, which is otherwise normal in her healthy mother [54].

## 5. Conclusions

Our case provides valuable clinical and pathophysiological insights. In fact, we present in a female carrier a novel form of NEMO deficiency in the absence of IP, which marks up an intriguing link between immunodeficiency and inflammation. Further, we provide an example of the expanding spectrum of NEMO disorders with critical counseling implications. 

## Figures and Tables

**Table 1:** Immunological values.

	Frequency (%)	Normal Range
CD3+CD45+	80	62–81
**CD19+CD45+**	5	6.5–24.0
NK (CD3-CD16+CD56+)	13	6–23
**% T lymphocyte subsets**		
CD3+CD4+	49	31–53
CD3+CD4+CD27+CD45RA+ Naïve	45.6	31–57
**CD3+CD4+CD27+CD45RA- Central memory**	**43**	10–27
**CD3+CD4+CD27-CD45RA- Effector memory**	**10**	12–44
**CD3+CD4+CD27-CD45RA+ EMRA**	0.32	4–12
CD3+CD4+CD31+CD45RA+ RTE	24.3	7–100
CD3+CD8+	19	19–30
CD3+CD8+CCR7+CD45RA+ Naïve	47.8	18–61
**CD3+CD8+CCR7+CD45RA- Central memory**	**2.2**	3–12
CD3+CD8+CCR7-CD45RA- Effector memory	37.2	25–58
CD3+CD8+CCR7-CD45RA+ EMRA	12.8	5–20
CD3+TCRαβ	86.4	36–98
TCRγδ	12.6	0.83–11
CD3+CD4-CD8-TCRαβ+	1.8	0.57–5
CD4+CD25hiCD127lowFoxp3+ Treg cells	6.2	3.7–9.0 (25–75° p.ile)
%CD3+CD4+ memory IL17+	1.76	0.27–4.1 (5–95° p.ile)
CD4+CD45RO+CXCR5+	10	8.3 ± 1.8% of CD4^+^ *
**% B lymphocyte subsets (on CD19+)**		
CD27+IgD+IgM+ Unswitched memory	7.6	2.6–13.4
**CD27+IgD-IgM- Switched memory**	25.5	4.0–21.2
CD27-IgD+IgM+ Naïve	63.6	61.6–87.4
CD27-IgD-IgM- Double negative	3.3	1.4–13.0
CD21low CD38low	3.6	3.2–19.6
**CD24high CD38high Transitional**	**0.73**	0.9–5.7
CD38++IgM- PlasmaBlasts	0.7	0.4–2.4
**Serum immunoglobulin levels**		
Serum IgG mg/dL	1101	604–1909
Serum IgM mg/dL	177	59–297
**Serum IgA mg/d**L	**392**	61–301
**Serum IgE UI/m**L	**208**	<100°

B cell subsets: Piatosa B. et al. Cytometry part B (Clinical Cytometry) 2010 and Duchamp M. et al. Immunity, Inflammation and Disease 2014. T cell subsets: Garcia-Prat M. et al. Cytometry B Clin Cytom. 2019; Schatorje’ E. J. H. Clinical Immunology 2012 and * Morita R. et al. Immunity. 2011. Serum Immunoglobulin concentrations: Italian Pediatric Hematology Oncology Association (AIEOP) https://www.aieop.org/web/. °IgE internal laboratory values.

## Data Availability

The raw data supporting the conclusions of this article will be made available by the authors on request.

## Supplementary Materials

The following supporting information can be downloaded at: https://www.mdpi.com/article/10.3390/jcm14020363/s1, Table S1: Excluded predicted variants from WES; Table S2: Negative gene for Mendelian susceptibility to mycobacterial diseases.
